# Cigarette smoking exposure disrupts the regenerative potential of dental pulp stem cells

**DOI:** 10.18332/tid/168125

**Published:** 2023-08-01

**Authors:** Leyla Tahrani Hardin, David Vang, Der Thor, Xiaoyuan Han, Fatima Mashkoor, Tamer Alpagot, David M. Ojcius, Nan Xiao

**Affiliations:** 1 Department of Biomedical Sciences, Arthur A. Dugoni School of Dentistry, University of the Pacific, San Francisco, United States; 2 Department of Oral and Maxillofacial Surgery, Arthur A. Dugoni School of Dentistry, University of the Pacific, San Francisco, United States; 3 Department of Periodontics, Arthur A. Dugoni School of Dentistry, University of the Pacific, San Francisco, United States

**Keywords:** DPSCs, nicotine, smoking, regeneration, inflammation

## Abstract

**INTRODUCTION:**

Smoking is known to alter the regenerative and immunomodulatory properties of many types of mesenchymal stem cells (MSCs). This study investigates the impact of cigarette smoke exposure on the regenerative potential of dental pulp stem cells (DPSCs).

**METHODS:**

DPSCs were treated with various doses of cigarette smoke condensate (CSC) or nicotine. Cell proliferation and survival were evaluated by a water-soluble tetrazolium salt (WST-1) and a survival assay. DPSC migration, cytokine expression, mutagenesis, and the signaling pathway were also measured during CSC and nicotine treatment.

**RESULTS:**

Low concentrations of CSC and nicotine did not impair cell proliferation, but higher concentrations reduced cell proliferation. CSC and nicotine could impede DPSC survival and migration in a dose-dependent manner. In addition, the cytokine secretion expression profile was altered with CSC or nicotine treatments. In particular, secretion of IL-6, TNF-α, and IL-10 significantly increased, while TGF-β1 levels showed different patterns after exposure to CSC or nicotine, as shown by ELISA and quantitative PCR. Nicotine treatment increased AKT (also known as protein kinase B) and extracellular signal-regulated kinase (ERK) phosphorylation. Finally, CSC induced higher levels of mutagenicity than nicotine, as shown by the Ames test.

**CONCLUSIONS:**

These findings suggest that cigarette smoke exposure alters the regenerative abilities of DPSCs in various ways. Future studies are warranted to further characterize the underlying molecular mechanisms of smoking-mediated damage to DPSCs, which will guide the personalized stem cell treatment plan for smoking patients.

## INTRODUCTION

Cigarette smoking ranks as a major public health problem whose negative impacts have spread around the world. Cigarette smoke contains over 4500 chemical compounds, including free radicals and oxidants. These compounds are present in both the gas phase (cigarette smoke extract, CSE) and the tar phase (cigarette smoke condensate, CSC). The gas phase contains metals, a large number of oxidants, and short-lived free radicals, whereas the tar fraction comprises essentially carcinogenic chemicals and long-lived free radicals^1^. Nicotine concentration is higher in CSC than in CSE^2^.

While much is known about the effect of smoking on many systemic diseases, little is known about its effects on stem cell therapy. Cigarette smoke exposure impaired mice’s bone marrow mesenchymal stem cell (BMMSC) migration and proliferation^3^. Endometrial stem cell self-renewal, migration, and osteogenic and adipogenic differentiation were impaired after exposure to CSE^4^. Human adipose stem cells (ASCs) from smokers failed to improve perfusion in the ischemic limbs of the animals, and were less potent in supporting epithelial cell survival, proliferation, and angiogenesis. ASCs from non-smokers expressed significantly lower levels of stromal-derived growth factor (SDF-1) and hepatocyte growth factor (HGF) compared to cells from smokers^5^.

The cellular impact of nicotine varies depending on the dose of treatment and the cell type. Nicotine was reported to promote proliferation of mouse embryonic stem cells at 0.01 μM and 0.1 μM, but suppress proliferation at 1 μM and 10 μM^6^. Proliferation and migration of the bronchioalveolar lung carcinoma cell line A549 was stimulated by 1 μM of nicotine^7^. In LoVo and SW620 human colorectal cancer cell lines, nicotine (0.1, 1, and 10 μM) promoted migration^8^. Inhibition of migration was observed in gastric epithelial cells with much higher doses of nicotine (200 μg/mL)^9^.

Dental origin stem cells, such as dental pulp stem cells (DPSCs) and periodontal ligament stem cells (PDLSCs), have received more attention as sources of stem cells, because of their easy accessibility and comparable properties to BMMSC. There are discrepancies between different reports about the impact of smoking on dental stem cells. It was reported that the cell proliferation of the DPSCs from non-smokers was significantly higher than that of smokers^10^. Nicotine accelerated proliferation and increased matrix metalloproteinase (MMP)-2 and MMP-28 expression in DPSCs^11^. PDLSCs isolated from smokers presented reduced proliferation and migration capabilities when compared to cells from non-smokers^12^. Nicotine was also found to decrease human gingival fibroblast migration rates by 50% at 0.5 μM^13^. Migration of cementoblasts was reduced by more than 78.4% at 1 mM nicotine^14^. More research is warranted to valid the impact of nicotine and CSC on dental origin stem cells.

We investigated the impact of cigarette smoke on the regenerative properties of human DPSCs in this research.

## METHODS

### CSC and nicotine

CSC (reference 3R4F, 40 mg/mL, 1 mL/vial in DMSO) was obtained from Murty Pharmaceuticals (Lexington, Kentucky, USA), and diluted in culture medium to the working concentration. Nicotine was purchased from Sigma-Aldrich (Catalog# N3876) and dissolved in filtrated pure ethyl alcohol (EtOH).

### Cell culture and treatment conditions

The human DPSCs were purchased from Lonza (Catalog #PT-5025, Lonza, OR). DPSCs were cultured in alpha-modified minimum essential medium α-MEM (Fischer Scientific, IL) containing 10% fetal bovine serum (FBS) (Neuromics, MN), 1% antibiotics (penicillin-streptomycin), and glutamate solution. Passages from two to six were used in experiments.

### WST-1 cell proliferation assay

Water-soluble tetrazolium salt (WST-1) assay (Catalog #MK400, Takara Bio, CA) was performed following the manufacturer’s instructions. In brief, 1 × 105 DPSCs were seeded in 96-well plates and exposed to different concentrations of CSC or nicotine for up to 96 hours. Cells were then washed twice with PBS and incubated in fresh medium with 10% WST-1 reagent for 4 h. Absorbance was measured at 440 nm in a multiplate reader.

### Survival assay

DPSCs were seeded at 2×10^3^ and 2×10^4^ cells/well in 6-well plates with serum-free medium, treated with different concentrations of CSC or nicotine, and incubated for 10 days. Cells were fixed with methanol:acetic acid (7:1, vol/vol) and stained with crystal violet 0.8% for 15 min, then rinsed with water and air-dried at room temperature.

### Wound healing assay

In all, 1.5×10^5^ DPSCs were seeded into 12-well plates then serum starved overnight after reaching confluence. The cells were scratched with sterile pipette tips, washed with PBS, replenished with serum-free medium and exposed to various concentrations of CSC or nicotine for up to 48 h.

### Cytokines array and analysis

Membrane-based Cytokine Array C5 (Catalog #AAH-CYT-5, Raybiotech, GA) was used to compare expression levels of 80 human cytokines following the manufacturer’s instructions. Briefly, serum free media from DPSC culture (1.5×10^5^/well in a 12-well plate) were collected after 48-h exposure to CSC or nicotine. Concentrated samples were incubated with the cytokine array membrane for 5 h at room temperature or overnight at 4°C with shaking. Chemiluminescent images were acquired. The cytokine levels were first normalized to the positive control standard on each membrane. The fold change of cytokine levels in treated cells was calculated over the level of the same cytokines in the matched untreated cells.

### The enzyme-linked immunosorbent assay (ELISA)

Serum free medium collected from DPSC culture (1.5×10^5^/well in 12-well plate) was used to analyze human Il-6, IL-10, and TGF-β1 in supernatants using a sandwich ELISA kit (Catalog #DY206, DY217B, DY240-05, and DY210-05 from R&D systems) according to the manufacturer’s protocol.

### RNA isolation and quantitative real-time polymerase chain reaction (qRT-PCR) analysis

Qiagen RNeasy isolation kit (Catalog #ID74104, Valencia, CA) was used to isolate total RNA from DPSCs (1.5×10^5^/well in 12-well plate). A high-capacity cDNA reverse transcription kit (Catalog #4368813, Applied biosystems, Thermo Fisher Scientific) was used for cDNA synthesis. Sequences of the primers used in qRT-PCR can be found in Supplementary file Table 1.

### Western blot

Cell lysate was collected from DPSCs (5×10^5^/well in 6-well plate) treated with different doses of nicotine for 5 min and 1 h. Membranes were probed with rabbit anti-phospho-AKT (Ser473), rabbit anti-phospho-ERK and anti-β-tubulin (Catalog #9271, #9106S and #2128 Cell signaling technology, Danvers MA). Quantification was performed with Image J.

### Ames reverse bacterial mutagenicity test

Ames test was conducted on strain TA102 (Catalog #BAA-2722, ATCC, Manassas, VA) in the absence and presence of S9 activation. S9-cocktail contains 10% aroclor-induced Sprague-Dawley rat liver S9 post-mitochondrial supernatant (Catalog #Moltox 50 948 566, Fisher Scientific, IL)^15^. Approximately, 109 bacteria/mL were mixed with sodium phosphate buffer or a 10% S-9 mix as previously reported^16^. The positive controls were mitomycin C (0.5 μg/plate) in the absence of S9, and 2-aminoanthracene (5 μg/plate) in the presence of S9. Revertant colonies were counted after 72 h of incubation at 37°C.

### Data analysis

Image J was used for analyzing the acquired pictures. GraphPad Prim 9 software (GraphPad Software, Inc., La Jolla, CA) was used for statistical analysis using the Student’s t-test (2-tails) and ANOVA test (for multiple comparisons); differences with p values of <0.05 were considered significant. The CSC-treated samples were compared to culture medium-treated cells as controls, and the nicotine-treated samples were compared to EtOH-treated cells.

## RESULTS

### CSC and nicotine impair DPSC proliferation, survival and migration

**Figure 1:**
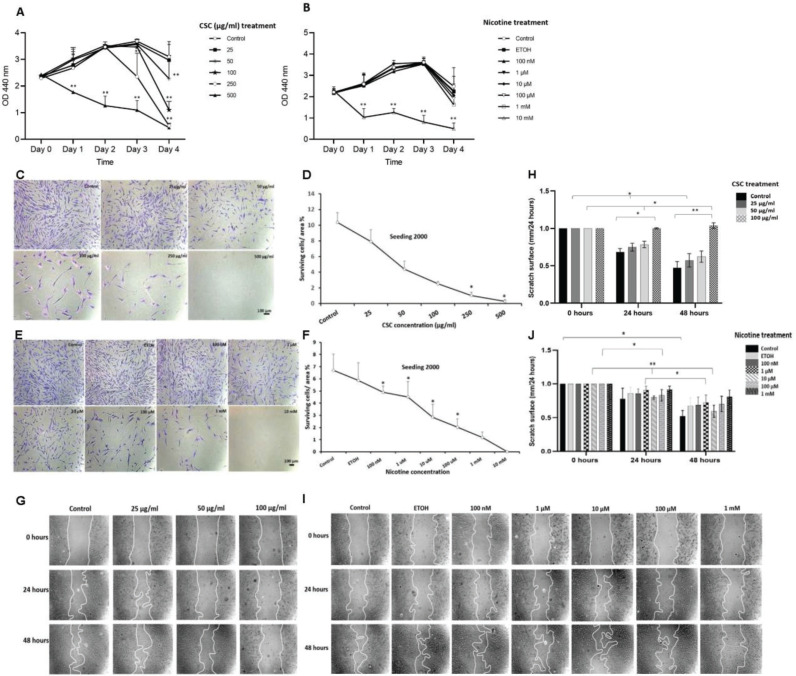
Proliferation, survival and migration of DPSCs after CSC and nicotine treatment. A) DPSCs were treated with different concentrations of CSC (25, 50, 100, 250 and 500 μg/mL); B) nicotine (100 nM, 1 μM, 10 μM, 100 μM, 1 mM and 10 mM) for four days; C) and D) DPSCs were seeded at 2000 cells per well and treated with CSC or nicotine; E) and F) quantification of the surviving cell covered area; G) and I) DPSCs were treated with CSC or nicotine for 48 h. Phase-contrast pictures of the wounds were taken at 0, 24, and 48 h; H) and J) quantification of the scratch surface area. ANOVA two-way test or Student’s t-test (two tails), CSC treated samples compared to control and nicotine treated samples compared to ETOH-treated vehicle control, *p<0.05, **p<0.01, n=3 experiments

To examine the potential toxic effects of CSC and nicotine on DPSCs, we performed an in vitro viability assay. WST-1 assay revealed that low concentrations of CSC did not impede DPSC proliferation for up to 3 days of treatment. On day 3, the 250 μg/mL CSC treatment induced a decrease in cell growth. On day 4, only the 25 μg/mL CSC-treated cells showed comparable cell growth as the control. The highest CSC concentration used (500 μg/mL) significantly (p<0.01) inhibited DPSC-growth starting from day 1. All the doses of nicotine treatment, except for the highest concentration (10 mM), did not impair normal cell growth for up to 3 days when compared to control or EtOH-treated cells. We included EtOH as a vehicle control in the study, since it was used for dissolving nicotine. By day 4, proliferation slowed down in all samples, which could be due to nutrient deficiency in the culture medium (Figures 1 A and B).

CSC and nicotine treatments also reduced the cell survival of DPSCs. Cell survival decreased in a dose-dependent manner in both CSC- and nicotine-treated cells. A similar pattern was found at low seeding numbers (2000 cells) (Figures 1 C–F) or high seeding numbers (20000 cells) (Supplementary file Figure 1). High concentrations of CSC (500 μg/mL) and nicotine (10 mM) completely inhibited the cell survival of DPSCs and were excluded from other assays.

The wound healing assay revealed the inhibitory effect of CSC and nicotine on the migration of DPSCs. The cells treated with 100 μg/mL of CSC for 48 h showed a significant inhibition (p<0.01) of 100%; 10 and 100 μM of nicotine treatment showed significant inhibition of migration after 48 h. The inhibition of cell migration was dose-dependent for CSC, but not for nicotine. Nicotine treatment at 10 μM showed a less inhibitory effect compared to other doses (Figure 1G-J). CSC treatment at 250 μg/mL in serum-free medium significantly reduced the attachment of DPSCs to the culture plates and was excluded.

### CSC and nicotine change the profile of cytokine secretion in DPSCs

The Cytokine Array C5, which includes 80 human cytokines, was used to screen cytokine secretion by DPSCs. Analysis showed distinguishing patterns between CSC-treated and nicotine-treated DPSCs. CSC (100 g/mL) treatment for 48 h led to a significant decrease in levels of transforming growth factor (TGF)-β2, TGF-β3, light, tissue inhibitor matrix metalloprotwinase (TIMP)-1, and pulmonary and activation-regulated chemokine (PARC). (Supplementary file Figures 2 A and B). Nicotine (1 mM) treatment led to a significant increase of interleukin (IL)-5, IL-6, tumor necrosis factor (TNF)-α, TNF-β, macrophage migration inhibitory factor (MIF), macrophage colony-stimulating factor (M-CSF), macrophage-derived chemokine (MDC), and TIMP-2, and a significant decrease of fibroblast growth factor (FGF)-4 (Supplementary file Figures 2 C and D). Although not significant, TGF-β1 and IL-10 increased by 10% after nicotine treatment.

**Figure 2:**
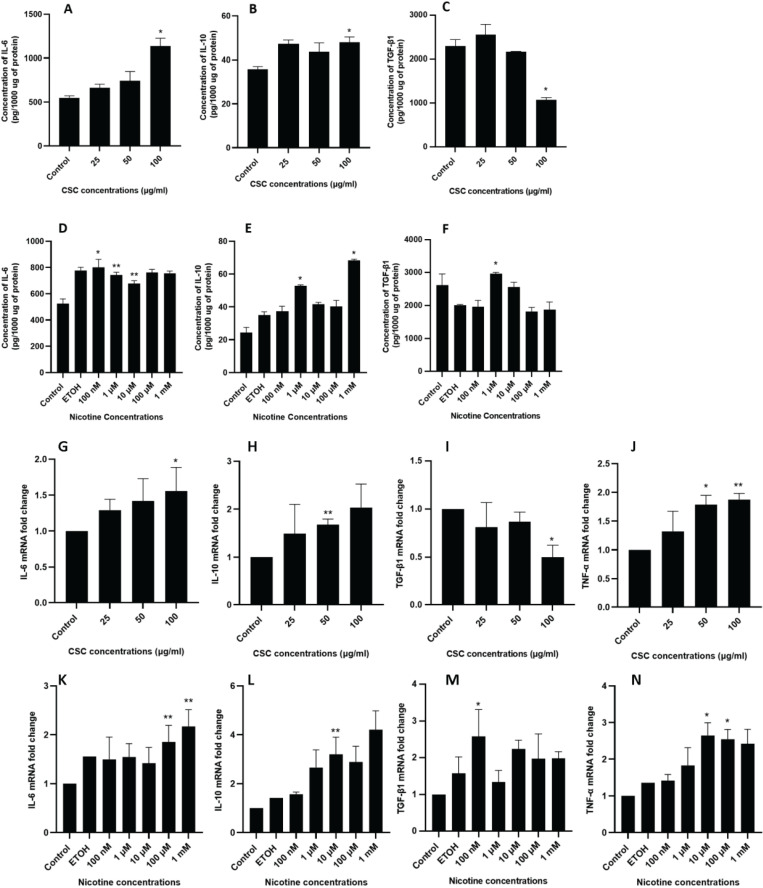
ELISA and qRT-PCR of cytokines in DPSCs after CSC and nicotine treatment. DPSCs were treated with CSC or nicotine for 48 h. ELISA quantification of cytokine concentration in serum free medium. Bars represent mean value. A) and D) IL-6; B) and E) IL-10; C) and F) TGF-β1. qRT-PCR of cytokine expression in cell lysate. Bars represent fold changes. G) and K) IL-6; H) and L) IL-10; I) and M) TGF-β1; J) and N) TNF-α. Student’s t-test (two tails), CSC treated samples compared to control and nicotine treated samples compared to ETOH-treated vehicle control, *p<0.05, **p<0.01, n=3 experiments

We further validate the changes of pro-inflammatory cytokines IL-6 and TNF-α, and anti-inflammatory cytokines IL-10 and TGF-β1. ELISA findings showed that both CSC and nicotine treatments induced an increased release of IL-6 (Figures 2 A and D) and IL-10 (Figures 2 B and E) from DPSCs in a dose-dependent manner. For TGF-β1, ELISA results showed a significant decrease at 100 μg/mL of CSC treatment (Figure 2 C) and a significant increase at 1 μM of nicotine treatments (Figure 2 F).

Similar expression profiles for IL-6 and IL-10 were confirmed through qRT-PCR (Figures 2 G, H, K and L). For TGF-β1, consistent with the ELISA results, 100 μg/mL of CSC treatment led to a significant reduction of expression (Figure 2 I), while 100 nM of nicotine treatment led to a significant increase of expression (Figure 2 M). In addition, both CSC and nicotine induced an increase of TNF-α cellular expression (Figures 2 J and N).

### Nicotine induces AKT and ERK phosphorylation

**Figure 3:**
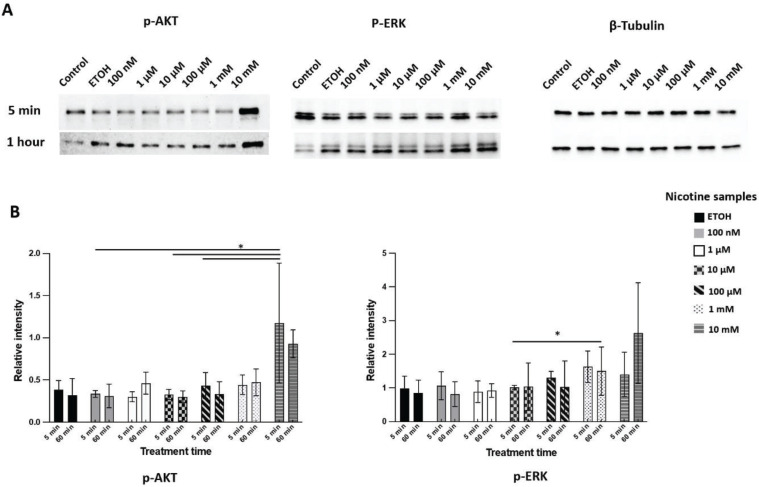
Expression of phosphorylated AKT (p-AKT) and ERK (p-ERK) in DPSCs after nicotine treatment. DPSCs were treated with culture medium (control), ethanol (ETOH), or different concentrations of nicotine (100 nM, 1 μM, 10 μM, 100 μM, and 1 mM) for 5 min or 1 h treatment. A) western blot images for phosphor-AKT S473, phosphor-ERK T202 Y204 and β-tubulin; B) quantification of chemiluminescent signal of western blots using image J software. Bars represent mean values after normalization. ANOVA two-way test, *p<0.05, n=3 experiments

Western blot showed phospho-AKT (Ser473) (also known as protein kinase B) increased after 5 minutes of nicotine treatment compared to the EtOH treatment, while phospho-ERK (extracellular signal-regulated kinase) increased after one hour of exposure (Figure 3). The results indicated a dose-dependent increase in AKT and ERK phosphorylation after one hour of exposure to different concentrations of nicotine. However, CSC treatment did not induce consistent activation of either phospho-AKT or phospho-ERK.

### CSC and nicotine induce mutagenesis

**Figure 4:**
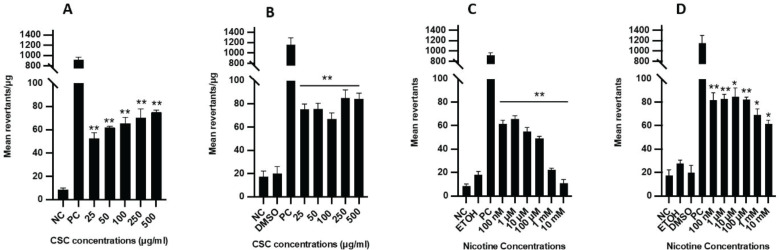
Ames reverse bacterial mutagenicity assay after CSC and nicotine treatment. The mean revertants per plate after CSC treatment: A) without or B) with the metabolic S9 fraction. The mean revertants per plate after nicotine treatment: C) without or D) with the metabolic S9 fraction. NC: negative control (autoclaved distilled water). PC: MMC without S9 and 2-aminoanthracene with S9. Vehicles: ETOH and DMSO. Bars represent mean values. Student’s t-test (two tails), *p<0.05, **p<0.01, n=3 experiments. CSC treated samples were compared to control (culture medium-treated cells) and nicotine treated samples were compared to ETOH-treated cells (vehicle control)

In the mutagenesis assay, the specific activity for all CSC and nicotine concentrations examined ranged 47–79 and 8–67 revertants/plate, respectively (Supplementary file Figure 3). S9 fraction was added to activate the cellular metabolic reaction that is needed to trigger the mutagenic activity of certain substances. A dose-dependent increase in mutagenicity was observed in the samples treated with CSC with or without the S9 fraction, and a small increase in Ames activity was observed for CSC with the addition of the S9 fraction (Figures 4 A and B). Interestingly, low concentrations of nicotine (100 nM – 100 μM) were more mutagenic than high concentrations (1–10 mM) without the S9 fraction (Figure 4 C). A remarkable increase in mutagenic activity was detected for nicotine treatment when the S9 fraction was added, especially at high concentrations (1–10 mM) (Figure 4 D).

## DISCUSSION

In the present study, we found that cigarette smoke exposure inhibited the regenerative abilities of DPSCs through changes in different biological properties. Compared to bone marrow mesenchymal stem cells (BMMSCs), DPSCs have higher angiogenic, neurogenic, and regenerative potentials^17^. The unique dental origin DPSCs are promising therapeutic targets in tissue regeneration and, more recently, in anti-inflammatory treatment because of their low immunogenicity.

We examined the impact of CSC and nicotine on DPSCs’ regenerative properties. The concentrations of CSC (25–500 μg/mL) and nicotine (100 nM – 10 mM) mimicked cigarette smoke exposure in humans. Previous studies reported that CSC contains 55.8% nicotine in addition to other components such as nicotyrine, 1,2,3-propanetriol, triacetate, ethyl chloride, and phenol^2^. The average steady-state serum concentrations of nicotine after smoking one cigarette, containing 0.8–1.9 mg of nicotine, have been reported to be 200 nM^18^, and increase to 10–100 μM in serum or to 1 mM at the mucosal surface immediately after smoking^19,20^. In this study, both low and high doses were used to mimic the smoking impact in light and heavy smokers.

Results showed that higher doses of CSC (250–500 g/mL) or nicotine (10 mM) inhibited DPSC proliferation. DPSC survival showed dose-dependent impairment after CSC or nicotine treatment, indicating that the damage from cigarette smoke probably occurred immediately after exposure and continued for a much longer period of time. The wound healing properties of DPSCs are more sensitive to cigarette smoke exposure. As low as 100 g/mL CSC or 1 mM nicotine significantly reduced DPSC migration. Wound healing is a typical condition in which cells are first subjected to activation of their motility in order to repopulate the damaged region, and then they show a strong proliferative response in order to successfully complete the wound repair process. Many factors play a critical role in regulating cellular function, such as cytokine concentration, which is the key determiner of cell behavior, plasticity, and robustness^21^.

DPSC culture medium contains a variety of cytokines such as IL-6, IL-10, IL-13, 1, TIMP-1, TIMP2, TGF-β, monocyte chemoattractant protein (MCP)-1, granulocyte-macrophage colony-stimulating factor (GM-CSF), osteoprotegerin, growth related oncogene (GRO), stromal cell-derived factor (SDF)-1, and hepatocyte growth factor (HGF)^22^. These cytokines represent key-messengers for immune and non-immune cell communications. In our study, we found different cytokine secretion profiles in DPSCs treated with CSC or nicotine. Among the cytokines whose expression was significantly altered after exposure to CSC or nicotine, TIMP was previously reported to mediate ECM degradation in vascular walls by inhibiting the activity of matrix metalloproteinases (MMPs) and was detected in the extracellular vesicles (EVs) from DPSCs^23^. IL-10 was reported to promote proliferation and migration, but inhibit differentiation of tendon-derived stem cells^24^. Both IL-6 and TNF-α are known to regulate pro-inflammatory responses and to influence angiogenic potential, which could be important in wound healing and fracture repair^25^. In addition, IL-6 induced proliferation in hepatocellular carcinoma cell lines which was attenuated by TGF-β^26^.

We found that CSC treatment led to a decrease in TGF-β1, while low dose nicotine treatment increased expression of the cytokine. TGF-β1 plays a crucial role in cell proliferation^27^, migration and dentin formation, and thus contributes to regeneration of the dentin–pulp complex^28^. A previous study reported that 25 μg/mL of CSC decreased TGF-β and enhanced tumorigenicity in lung cancer cells^29^. On the other hand, tobacco smoking (90 mg/m3 total suspended particulate) increased TGF-β in epithelial cells in rats^30^. The plasma levels of TGF-β1 were significantly elevated in smokers compared with healthy controls, and the level was related to the duration of cigarette smoking^31^. As TGF-β cascade crosstalk with many different pathways regulating cell proliferation, survival, apoptosis, autophagy, and senescence, it would be important to further study how the cytokine responds to cigarette smoke in animal models to understand its function in different settings. It is interesting that our results echo previous reports that nicotine showed pro- and/or anti-cell proliferation and migration effects in different cells at various doses and durations of exposure, indicating cell-specific mechanisms might be responsible for the differential consequences.

Inflammation plays a dynamic role in tissue regeneration. During the wound healing process, cytokines serve as important signaling mediators between stem cells and other types of cells. Only a few studies have investigated the immunomodulatory ability of DPSCs under inflammatory conditions. Transplantation of DPSCs reduced inflammation in neurodegenerative diseases, such as Alzheimer’s and Parkinson’s. DPSCs attenuate mitogen-induced lympho-proliferative responses, and priming DPSCs with either IFNγ or TNF-α enhanced the immuno-modulation capacity^32^.

Both the PI3K/AKT and MAPK/ERK pathways play crucial roles in multiple cellular processes such as cell-cycle progression, protection from apoptosis, differentiation, adhesion, motility and immunity. Activated AKT can regulate immune cell cytokine secretion and reactive oxygen species (ROS) production, and maintain cell homeostasis by regulating the balance between apoptosis and proliferation through glycogen synthase kinase^33^. We did not find a clear pattern of AKT or ERK phosphorylation after CSC treatment, but nicotine treatment induced phosphorylation of both AKT and ERK in a dose-dependent manner. Nonetheless, CSC showed a higher mutagenic effect than nicotine. The biological response to cigarette smoke and its involvement in many comorbidities are complex, the AKT and ERK pathways may help to understand the effect of cigarette smoke on cell cycle regulators, apoptosis mediators, angiogenic factors, and survival.

ROS might be a key link between the mutagenesis and inflammation induced by cigarette smoke^34^. The findings are consistent with previous reports that smoke-derived oxidants disrupted cell signaling pathways such as NF-κB, MAPK and AP-1, which triggered the activation of cytokine production, growth factor release, and transcription factor activation^34-36^.

## CONCLUSIONS

Our study indicates that cigarette smoking can alter DPSC regenerative potential by inhibiting cell proliferation, survival, and migration, altering cytokine secretion, and inducing mutagenesis. The AKT and/or ERK pathways might be key players in mediating these effects. The study helps to understand the impact of short-term exposure to cigarette smoke components on stem cells and the underlying molecular mechanism. DPSCs represent a promising therapeutic alternative in the cutting-edge research field of regenerative medicine applications. It is necessary to narrow the knowledge gap on DPSCs in relation to environmental factors and lifestyles to enhance the success of personalized stem cell therapy in patients with a history of smoking. On the other hand, the study is conducted through in vitro assays only, which would not fully reflect the complexity of the impact of smoking on stem cells in vivo. Although the AKT and ERK pathways regulate important cellular functions, they are subject to crosstalk with many other pathways, such as the STAT and the TGF-β cascade. The interaction of these pathways and their contribution to cellular activities in response to cigarette smoke remain to be further investigated. Future studies on the differential impact of CSC and nicotine on the cytokine expression profile and the signaling pathway are warranted to better understand the underlying mechanism.

## Data Availability

The data supporting this research are available from the authors on reasonable request.
