# Supplementary material for: Cigarette smoking exposure disrupts the regenerative potential of dental pulp stem cells
Source: Tob Induc Dis. 2023 Aug 1;21:101. doi: 10.18332/tid/168125 (PMC10392041; doi:10.18332/tid/168125)
Supplement: Supplementary file 1 [file TID-21-101-s1.pdf]

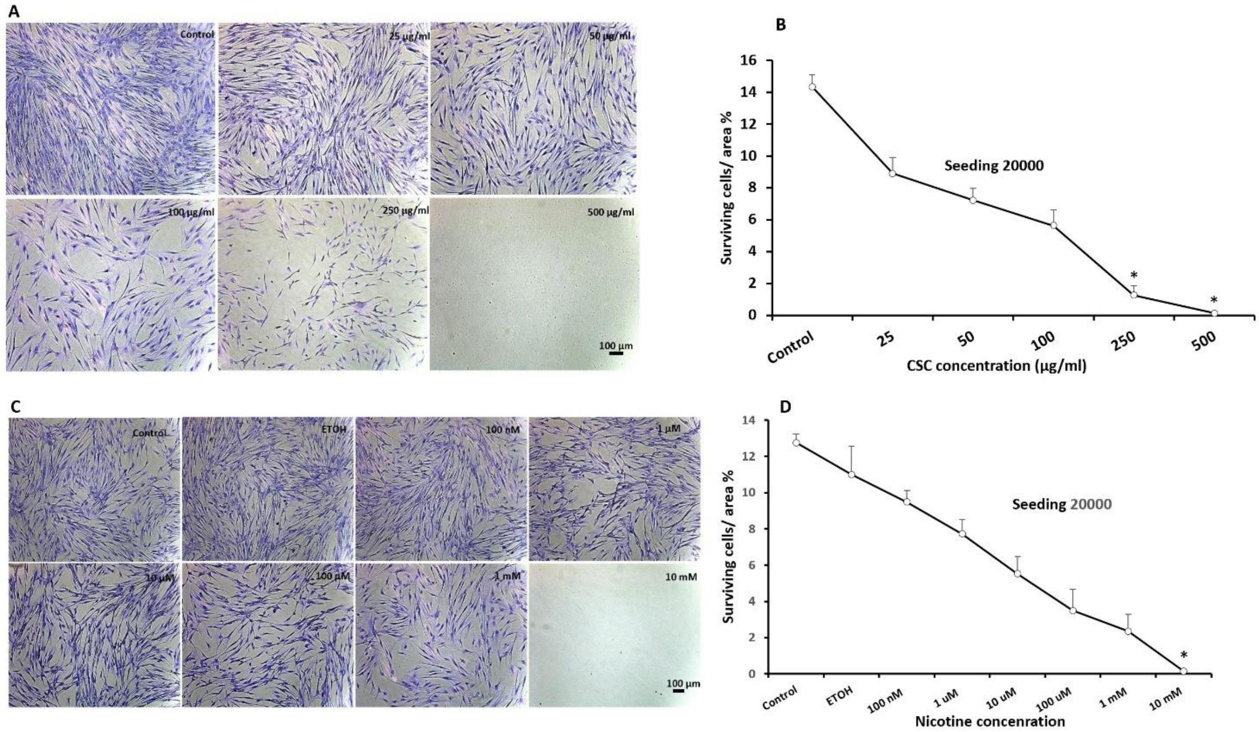

Supplementary Figure 1. Survival assay of DPSCs after CSC and nicotine treatment. DPSCs were seeded at 20,000 cells per well and treated with; **A**: 25 ug/ml to 500 ug/ml of CSC, or **B**: 100 nM to 10 mM nicotine. Test was conducted in triplicates. **B** and **D**: quantification of the colonies (10 pictures per treatment concentration were analyzed using Image J), ANOVA two-way test, $*p<0.05$, n=3 experiments.

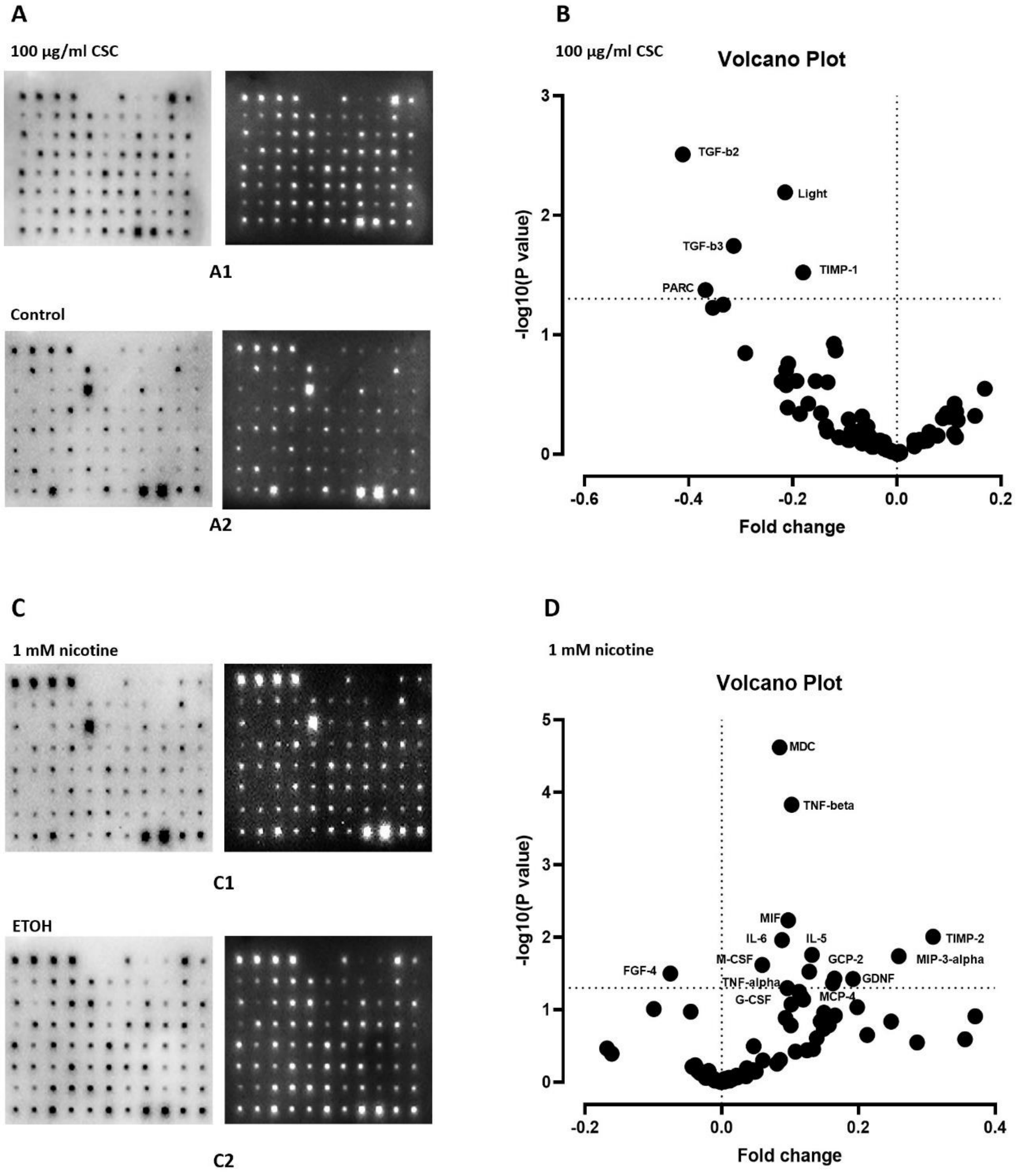

Supplementary Figure 2. Cytokines array of cell culture medium of DPSCs treated with 100 µg/ml of CSC or 1 mM nicotine for 48 hours; **A** and **C:** The C5 membranes; Dots quantification (3 pictures per treatment concentration were analyzed using Image J), $*p<0.05$. **B** and **D:** the Volcano plots, the x-axis shows fold change for each cytokine, The y-axis shows the variable importance of change (-log10(p value). Cut-offs of fold change over 0.5 and under -0.5, and -log10(p value) >1, were used and are illustrated by dotted lines. n=3 experiments.

CSC exposure

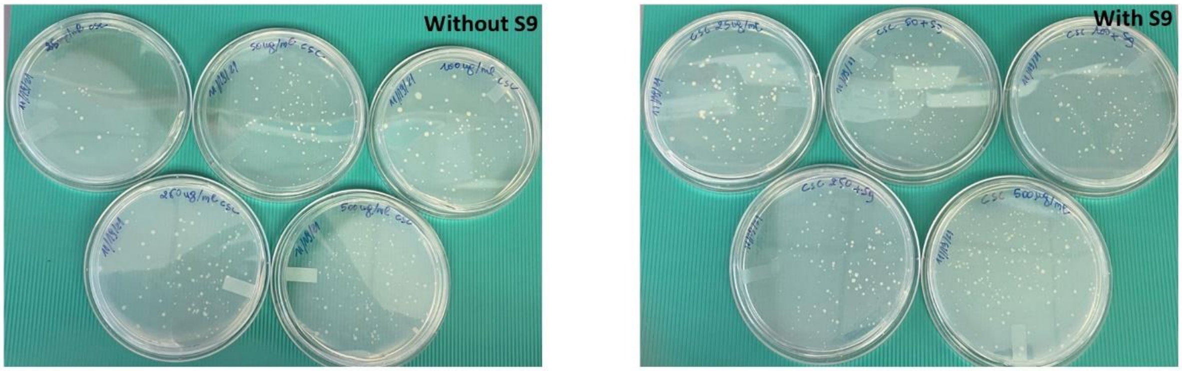

Nicotine exposure

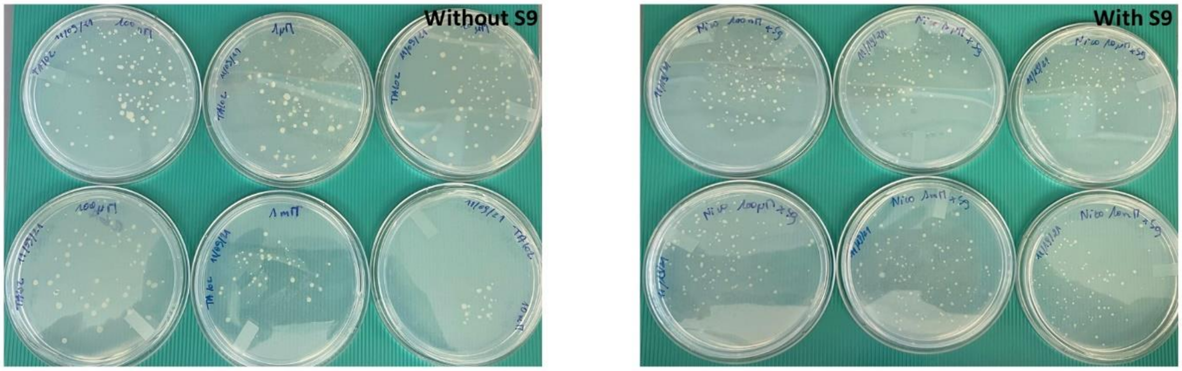

Supplementary Figure 3. AMES test showing the revertants after CSC and nicotine exposures. TA102 was exposed to different concentrations of CSC (25, 50, and 100, 250, and 500 µg/ml), or nicotine (100 nM, 1, µM, 10 µM, 100 µM, and 1 mM), in the absence and the presence of S9 activation.

Table S1: List of qPCR primers.

Gene	Forward primers	Reverse primers
B-actin	AGAGCTACGAGCTGCCTGAC	AGCACTGTGTTGGCGTACAG
IL-6	ACTCACCTCTTCAGAACGAATTG	CCATCTTTGGAAGGTTCAGGTTG
IL-10	GACTTTAAGGGTTACCTGGGTTG	TCACATGCGCCTTGATGTCTG
TGF-β1	TACCTGAACCCGTGTTGCTCTC	GTTGCTGAGGTATCGCCAGGAA
TNF-α	CCCCAGGGACCTCTCTCTAATC	GGTTTGCTACAACATGGGCTACA

© **2023 Tahrani Hardin L. et al.**
